# The Modulation of Adipokines, Adipomyokines, and Sleep Disorders on Carcinogenesis

**DOI:** 10.3390/jcm12072655

**Published:** 2023-04-02

**Authors:** Anna Brzecka, Helena Martynowicz, Cyryl Daroszewski, Maciej Majchrzak, Maria Ejma, Marta Misiuk-Hojło, Siva G. Somasundaram, Cecil E. Kirkland, Monika Kosacka

**Affiliations:** 1 Department of Pulmonology and Lung Oncology, Wroclaw Medical University, Grabiszyńska 105, 53-439 Wroclaw, Poland; 2 Department of Internal and Occupational Diseases, Hypertension and Clinical Oncology, Wroclaw Medical University, Borowska 213, 50-556 Wroclaw, Poland; 3 Department of Thoracic Surgery, Wroclaw Medical University, Ludwika Pasteura 1, Grabiszyńska105, 53-439 Wroclaw, Poland; 4 Department of Neurology, Wroclaw Medical University, Borowska 213, 50-556 Wroclaw, Poland; 5 Department of Ophthalmology, Wroclaw Medical University, Borowska 213, 50-556 Wroclaw, Poland; 6 Department of Biological Sciences, Salem University, 223 West Main Street, Salem, WV 26426, USA

**Keywords:** adipokines, adipomyokines, obesity, sarcopenia, sleep disorders, cancer

## Abstract

Obesity and sarcopenia, i.e., decreased skeletal muscle mass and function, are global health challenges. Moreover, people with obesity and sedentary lifestyles often have sleep disorders. Despite the potential associations, metabolic disturbances linking obesity, sarcopenia, and sleep disorders with cancer are neither well-defined nor understood fully. Abnormal levels of adipokines and adipomyokines originating from both adipose tissue and skeletal muscles are observed in some patients with obesity, sarcopenia and sleep disorders, as well as in cancer patients. This warrants investigation with respect to carcinogenesis. Adipokines and adipomyokines may exert either pro-carcinogenic or anti-carcinogenic effects. These factors, acting independently or together, may significantly modulate the incidence and progression of cancer. This review indicates that one of the possible pathways influencing the development of cancer may be the mutual relationship between obesity and/or sarcopenia, sleep quantity and quality, and adipokines/adipomyokines excretion. Taking into account the high proportion of persons with obesity and sedentary lifestyles, as well as the associations of these conditions with sleep disturbances, more attention should be paid to the individual and combined effects on cancer pathophysiology.

## 1. Introduction

Adipose tissue acts like an endocrine organ, participating in many physiological processes [1]. Proteins excreted solely by adipocytes or by adipocytes and other cells are called adipokines [2]. Moreover, skeletal muscles are a source of a large family of peptides that exhibit endocrine and paracrine/autocrine effects denominated as myokines [3]. Some of the proteins excreted by adipocytes and myocytes are called adipomyokines [4]. There is a close interaction between myokines and adipokines [5].

Obesity is associated with altered adipokine production because of processes accompanying adipocytes’ hypertrophy and hyperplasia, such as chronic low-grade inflammation, fibrosis, and matrix remodeling [6]. Sarcopenic obesity can be diagnosed in a person with a BMI ≥ 30 kg/m^2^ and decreased muscle mass and function [7]. Loss of muscle mass and function, i.e., sarcopenia, as well as sarcopenic obesity, change the levels of myokine and adipokines [8]. Sarcopenic obesity may be further differentiated into sarcopenic subcutaneous and sarcopenic visceral obesity [9]. Among cancer patients, sarcopenic obesity occurs in about 25% of obese patients (5.9–39.2%) [10]. As there are important influences of both obesity and sarcopenia on cancer, the concept of sarcopenic obesity has been proposed as a specific pathophysiological entity [11].

Sleep disorders are frequently associated with obesity and sarcopenia. Sleep duration and quality influence muscle strength [12]. In community-dwelling adults (≥65 years old), short sleep duration (<6 h) was associated with both an almost 3-fold increased risk of sarcopenia and an almost 2-fold increased risk of obesity [13]. There is a strong correlation between sleep disorders and obesity, as well as a bidirectional relation between these two factors because obesity, with its consequences, frequently impairs sleep quality [14].

Sarcopenia and/or obesity frequently accompany cancer. Sarcopenia is associated with the diagnosis and poorer prognosis of cancer [15,16]. Obesity is a risk factor for some types of cancer [17]. The prevalence of sarcopenic obesity in cancer patients is high, reaching up to one-fifth–one-third of cancer patients, and is associated with poor survival [18,19]. However, there is a lack of clear evidence of the influence of sarcopenic obesity on cancer occurrence [20].

The role of adipokines and adipomyokines in cancerogenesis is controversial and has not been fully understood. Thus, taking into account that obesity, sarcopenia and sarcopenic obesity are all associated with both sleep disorders and cancer, as well as with disturbed adipokines/myokines concentrations, we aimed to trace the relationship between adipokines and one of the myokine, i.e., irisin, with sleep disturbances and cancer.

## 2. The Association of Sarcopenia, Obesity, and Sarcopenic Obesity with Cancer

Sarcopenia existing before a cancer diagnosis is an independent risk factor for several cancers, such as lung cancer, colorectal cancer, breast cancer, head and neck cancer, pancreatic cancer, gastric cancer, esophageal cancer, ovarian cancer and hepatocellular cancer [21]. A meta-analysis of the results of 38 studies encompassing 7843 patients with various solid tumors revealed that sarcopenia–as diagnosed on the results of computed tomography studies–was associated with a 44% lower overall survival (HR = 1.44, 95% CI = 1.32–1.56, *p* < 0.001) and a 95% lower cancer-specific survival (HR = 1.93, 95% CI = 1.38–2.70, *p* < 0.001) [22]. A systematic review of the studies on sarcopenia diagnosed by bioelectrical impedance analysis indicated adverse clinical outcomes of sarcopenia in cancer patients [23]. Another meta-analysis found that sarcopenia, assessed by computed tomography, was associated with worse overall survival in cancer patients [24]. Among cancer patients receiving immunotherapy, those with sarcopenia alone, as well as those with sarcopenia and biochemical signs of systemic inflammation, obtained a shorter overall survival rate (HR 4.01, 90% CI 1.66–9.68, *p* = 0.002 and HR 8.46, 90% CI 2.65–27.01, *p* < 0.01, respectively) and shorter progression-free survival (HR 2.14, 90% CI 1.12–4.10, *p* = 0.22 and HR 12.29, 90% CI 5.15–29.32, *p* < 0.001, respectively) [25]. In contrast, another review reported that low muscle mass was not a factor that negatively affected survival rates in incurable cancer; but the studies included in this meta-analysis did not encompass assessments of either muscle strength or physical function [26].

An association between obesity (or simply being overweight) and several cancers has been established, including colorectal, gallbladder, pancreas, kidney, endometrial, breast cancer in postmenopausal women, ovarian, gastric, cardiac, thyroid, esophageal, adenocarcinoma, and multiple myeloma [27]. The strongest associations between being overweight and cancer were found for endometrial cancer, esophageal carcinoma, and kidney cancer [28]. In 2012, 3.6% of newly diagnosed adult cancers could be attributed to being overweight or obese [29]. About 14% of cancer deaths in men and around 20% of cancer deaths in women likewise can be attributed to obesity [30].

Obesity leads to chronic, low-grade inflammation by a disturbed balance between adipokines and cytokines, such as–among others–interleukins (IL-6, IL-8), monocyte chemoattractant protein-1 or tumor necrosis factor-α [31]. Chronic inflammation associated with obesity may promote cancerogenesis through its influence on hormonal balance with further impact on the immune system [32] by inducing tumor cell proliferation and angiogenesis, as well as by damaging genetic material [33].

Metabolic disturbances associated with obesity may strongly influence neoplastic disease [34]. However, at the molecular level, a precise link between increased adipose tissue and cancer has not been fully explained [35].

Considering the variety of possible links between obesity and cancer, the type of obesity should be taken into account [36]. Obesity can be regarded as metabolically healthy, metabolically unhealthy (or abnormal), or sarcopenic [37]. Metabolically abnormal obesity, contrary to metabolically healthy obesity, is associated with the co-occurrence of metabolic syndrome, type 2 diabetes, hypertension, or cardiovascular-cerebrovascular diseases. There is no clear cut-off point between healthy and unhealthy obesity [38]. Metabolically healthy obesity may describe up to 30% of obese people [39].

Sarcopenic obesity is associated with poor outcomes in most cancer patients, regardless of the localization and method of treatment [10]. This has been found by multiple studies and confirmed by meta-analyses [22,40,41]. Recent findings revealed a shortened survival in patients with sarcopenic obesity and head and neck cancer [42,43], gastric cancer [44,45], pancreatic cancer [46,47,48,49], urothelial cancer [50], lymphoma [51], colorectal cancer [52], as well as in women with colorectal cancer [53]. A meta-analysis of 14 studies of cancer patients revealed negative clinical outcomes associated with sarcopenic obesity, such as increased drug toxicity, more frequent surgical complications, and shortened survival [40]. In patients with hepatocellular carcinoma undergoing a hepatectomy, sarcopenic obesity was associated with worse median survival than non-sarcopenic non-obese patients [54]. Visceral adiposity with low muscularity was a risk factor for decreased survival [55].

In contrast, another study reported that sarcopenic obesity was not a risk factor for surgically treated hepatocellular carcinoma [56]. In patients with esophago-gastric cancer who were receiving palliative chemotherapy, sarcopenic obesity was associated with the occurrence of neurotoxicity from the chemotherapeutics but not with the progression of the disease or survival [57]. In patients with non-small-cell lung cancer undergoing chemoradiotherapy, sarcopenic obesity, diagnosed in 14% of patients, did not influence survival [58]. Sarcopenic obesity was not associated with overall survival in breast cancer patients [59] or colorectal metastatic cancer patients [60].

The role of adipokines in promoting genomic instability linking obesity and cancer has not been fully elucidated [61]. Altered microRNA secretion in adipose tissue may be implicated in oncogenesis [62], e.g., the exposure of prostate cancer cells to leptin downregulated the expression of micro-RNA-628 and led to increased cancer cell proliferation [63]. It has been shown that the development of renal cell carcinoma might be associated with obesity-associated alterations in gene expression, such as DNA methylation, single nucleotide polymorphisms, histone modification and microRNAs [64]. In renal cancer cells, the high methylation in leptin receptors predicted an increased risk of cancer progression and shorter recurrence-free survival of renal cancer patients [65]. The leptin receptor gene variant rs1137101 was proposed as a possible risk factor for renal cell carcinoma [66].

**Figure 1:**
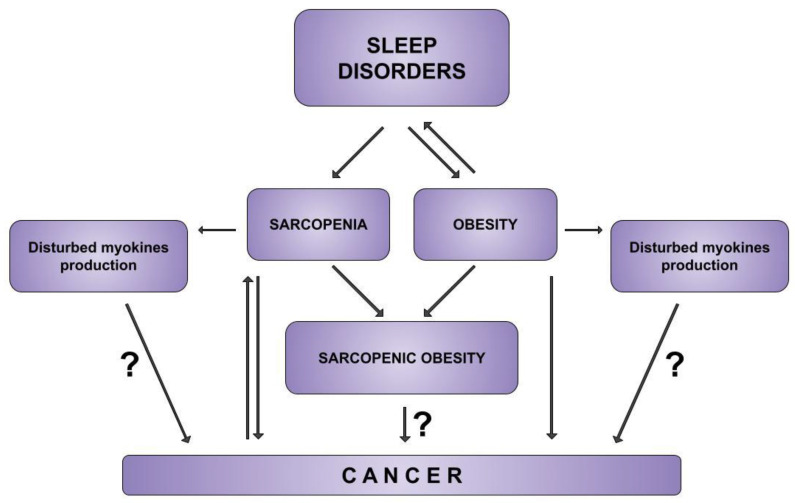
Possible association of sleep disorders, obesity and sarcopenia with cancerogenesis.

Possible associations between sleep disorders, obesity and sarcopenia with cancerogenesis are presented in Figure 1.

## 3. The Association of Sarcopenia, Obesity and Sarcopenic Obesity with Sleep Disorders

Age and inactivity [67,68] but also sleep disorders may influence progressive loss of muscle mass and function. Multiple sleep problems are associated with sarcopenia: long-sleep duration [69,70,71], disruption of sleep-wake rhythm by shift work [72], poor sleep quality [73], poor sleep quality in older patients with diabetes [74], increased sleep latency [75], later sleep timing [76], insomnia [77], poor sleep efficiency [78], complains of “problems sleeping” and taking sleeping pills [79]. As recently shown in the study encompassing 13,210 adults, long sleep duration (>9 h/day) in persons aged ≥ 65 years was significantly associated with sarcopenia, especially in women (odds ratio 2.19, 95% CI 1.26–3.81) [80].

Short- and long-sleep duration [81] and low sleep efficiency, especially in older men, were associated with obesity [82]. This association was reported by meta-analyses encompassing the result of studies of more than 5 million participants [83]. There is a bidirectional influence between sleep disorders increasing the probability of developing obesity and obesity increasing the chance of sleep disorders [84]. The most common sleep disorder associated with obesity is obstructive sleep apnea (OSA) [85].

Sarcopenic obesity may be associated with some sleep disorders, e.g., with OSA, that further worsens sleep quality [86].

## 4. The Association of Myokine Irisin with Sleep Disorders and Cancer

Irisin is the best-recognized myokine [87]; it is secreted by myocytes and adipocytes, and is also referred to as an adipomyokine [88]. Its levels depend on muscle mass and physical activity levels [89]. In obese patients, irisin levels are either decreased [8,90] or increased, indicating irisin resistance [91]. Irisin may be negatively influenced by sleep disturbances. Lowered irisin concentrations were associated with poor sleep quality, as shown in patients with rheumatoid arthritis [92]. OSA is often associated with obesity and generally leads to disordered sleep. Decreased irisin concentrations significantly and inversely correlated with OSA [93]. On the other side, however, elevated levels of irisin in OSA patients were associated with increased daytime sleepiness [94].

The role of irisin in carcinogenesis is not fully understood [95]. However, several recent findings indicate that irisin has a potent anticancerous action through different pathways [89]. Experimental studies have revealed that irisin suppressed the cell proliferation of many cancers, including pancreatic cancer cells [96], osteosarcoma cells [97], lung cancer cells [98], and breast cancer cells [99]. Irisin had no effect on the proliferation of cells linked to obesity-related cancers such as endometrial, colon, thyroid, and esophageal [100]. In pancreatic cancer cell lines, irisin activated adenosine monophosphate-activated protein kinase, downregulated the mTOR pathway and inhibited epithelial-to-mesenchymal transition leading to the suppression of the cell growth [96]. It was shown to inhibit endothelial-to-mesenchymal transition, a hallmark of cancer, via the STAT3/Snail signaling pathway in osteosarcoma [97]. In liver cancer cells, however, irisin was found to activate the PI3K/AKT pathway facilitating cancer progression [101].

Increased irisin serum levels correlated with a decreased risk of breast cancer [102] and colorectal cancer [103]. In one of the first clinical studies on the role of irisin in cancer patients, irisin levels in the serum of breast cancer patients were significantly lower than in the serum of healthy women and were associated with the tumor stage [102]. Additionally, irisin was found to play a protective role against spinal metastases in breast cancer patients. In patients with metastases to the spine, the concentrations of irisin in the serum were lower than in the patients without spinal metastases [104]. Decreased irisin serum levels were found in hepatocellular carcinoma patients [105]. A low irisin serum concentration may be regarded as a highly sensitive (80.5%) and specific (90%) biomarker of prostate cancer [106]. Low irisin serum concentrations in bladder cancer appeared to have high sensitivity (74.7%) and specificity (90.7%) as a diagnostic biomarker and predicted higher mortality rates in this type of cancer [107]. On the contrary, in renal cancer patients, irisin levels in the serum were higher than in the healthy controls [108]. Moreover, in patients with benign breast tumors or with breast cancer, irisin concentrations were elevated [109].

## 5. The Association of Adipokines with Sleep Disorders and Cancer

One adipokine that is negatively associated with obesity is adiponectin, an established anti-carcinogen. Another adipokine negatively associated with obesity is omentin-1, which has anti-inflammatory properties and is not clearly associated with carcinogenesis. Other adipokines that are positively correlated with obesity (leptin, resistin, vaspin, chemerin, nesfatin) do not exhibit an equivocal role in carcinogenesis. Some adipokines that are positively correlated with obesity (visfatin, osteopontin, apelin, retinol-binding protein 4, galectin) exert pro-carcinogenic effects. All these adipokines are influenced by sleep disorders.

### 5.1. Adiponectin

Adiponectin has an inverse correlation with BMI [110]. Adiponectin serum concentrations are significantly decreased in OSA [111,112,113,114]. Improved quality of sleep with the treatment of OSA resulted in increases in adiponectin serum concentrations [115]. In other situations of sleep loss, decreased levels of adiponectin were found [116], although this observation has not been confirmed in other studies [117,118].

Adiponectin is considered an adipokine with anti-tumor properties [119]. Hypoadiponectinemia is associated with an increased risk of various cancers, as shown by several meta-analyses [120,121,122].

### 5.2. Omentin-1

There is an inverse correlation between omentin-1 and obesity [123]. Omentin plasma levels were found to be significantly decreased in OSA patients [112] and correlated with sleep structure abnormalities [124].

In vitro studies of omentin-1 indicated both a potentially carcinogenetic role [125] and a protective role against cancer [126]. The meta-analysis revealed that increased levels of omentin-1 were strongly associated with an increased risk of colorectal, pancreas, and breast cancers [127]. The association between increased omentin plasma levels and colorectal cancer was found only in non-obese patients, with no relationship in obese colorectal cancer patients [128]. Increased concentrations of omentin-1 plasma levels were found in prostate cancer patients, and it has been postulated that its levels could serve as a diagnostic biomarker in this type of cancer [129]. A meta-analysis of case-control studies found downregulation of omentin in patients with lung cancer [130] and significantly decreased serum levels of omentin-1 in breast cancer patients [131] or it’s serum levels in renal cancer patients were found [132]. In breast cancer postmenopausal patients, omentin-1 serum levels were inversely associated with tumor markers and cancer stage [133].

### 5.3. Leptin

Leptin concentrations are positively correlated with obesity [1]. Leptin serum levels are increased in OSA syndrome patients, mostly because of obesity [112,134,135,136], and, in some studies, it correlated with the severity of this syndrome [137]. Moreover, poor sleep quality in overweight and obese subjects without OSA was associated with increased levels of leptin [138]. Short sleep duration led to an increase in leptin serum levels [139].

Leptin favors cancer cell proliferation and invasion, influencing cancer cell differentiation and migration, stimulating angiogenesis, and inhibiting cancer cell apoptosis [140]. The influence of leptin plasma levels on the development of cancer has been established in patients with breast cancer [141] and endometrial cancer [142]. In paraffin blocks taken from patients with colorectal cancer, an increased expression of leptin receptors was found [143]. In patients with glioma, the expression of leptin and leptin receptors in the specimens of resected tumors significantly correlated with the level of malignancy [144]. In patients with meningioma, the expression of leptin receptors in the tumor positively correlated with BMI [145]. In patients with ovarian cancer, leptin serum levels did not correlate with the stage of the disease [146].

### 5.4. Resistin

Resistin has a known relationship with obesity [147]. In one early study, resistin serum levels in OSA patients were found to be decreased [134]. Another study did not confirm the association between OSA and resistin levels [148]; however, recent reports indicated that OSA was associated with increased resistin serum concentrations [112].

Although some previous studies and meta-analyses have reported a positive correlation between increased resistin levels and cancer [149,150,151,152], another meta-analysis did not confirm a relationship between resistin and cancer risk [153].

### 5.5. Vaspin

Vaspin serum concentrations were found to be increased in obesity [154]. Vaspin plasma levels were increased in severe OSA patients compared to the controls [155].

Vaspin serum concentrations were significantly elevated in hepatocellular carcinoma, especially in obese patients [156]. However, decreased vaspin serum concentrations were associated with an increased risk of endometrial cancer in patients [157].

### 5.6. Chemerin

Chemerin is positively associated with obesity [158,159]. Chemerin’s serum levels are increased in OSA patients [112,155] and correlate with the severity of the disease; they were postulated to serve as a biomarker of the presence and severity of OSA syndrome [160].

Increased circulating levels of chemerin were found in non-small-cell lung cancer, tongue, esophageal, gastric, and colorectal cancers, as well as in neuroblastoma [140]. However, there are controversies regarding its role in cancer development, as its anti-tumoral and pro-tumoral actions have been described [161].

### 5.7. Nesfatin

Circulating nesfatin-1 levels positively correlate with BMI in humans [162]. The function of nesfatin-1 is related to energy homeostasis, behavior and sleep [163]. In patients with major depression, nesfatin-1 was found to be involved in the regulation of sleep patterns [164]. In OSA patients, nesfatin-1 serum levels were decreased, compared to healthy controls, and correlated with the severity of this syndrome [165,166], although not all studies confirm such associations [167].

Nesfatin was found to be elevated in colon cancer tissue [168]. In patients with gastric cancer, its plasma levels have been proposed as a novel biomarker [169]. Conversely, in lung cancer patients, nesfatin-1 serum levels depended on weight loss and either were not different from healthy controls or decreased based on the patients and weight loss [170].

### 5.8. Visfatin

Visfatin serum concentrations positively correlate with BMI [171]. Increased plasma levels of visfatin were found in OSA patients [172], although not all studies confirm this observation [124]. In one study, visfatin plasma levels in severe OSA, although not different than in the controls, correlated positively with disturbances in sleep architecture [173]. Interestingly, circulating visfatin levels were significantly increased in patients with narcolepsy: a disease associated with profound sleep architecture disturbances [174]. Similarly, short sleep duration and disturbed sleep architecture (short REM sleep duration) in patients without OSA were associated with increased visfatin serum levels [139].

A recent meta-analysis of 14 studies encompassing 1616 patients revealed increased expression of visfatin in various cancers and the association of this adipokine with poorer overall survival, as well as with tumor size, tumor stage, and the presence of lymph nodes or distant metastases [175].

### 5.9. Osteopontin

Osteopontin levels increase in obesity [176]. Osteopontin plasma levels were not different in patients with and without OSA but increased with OSA severity and daytime sleepiness [177].

Osteopontin plays an important role in tumor progression, promoting, among other things, tumor growth and tumor cell invasion [178]. Increased expression of osteopontin was found in human bladder cancer cell lines [179]. Increased plasma levels of osteopontin were found in melanoma patients, especially in patients with metastases [180]. It was found in the cell lines that cancer cell motility was regulated by osteopontin secreted by the cancer cells [181].

### 5.10. Apelin

Apelin levels increase in obese patients [182]. Plasma apelin levels in OSA patients were found to either be increased [183] or not influenced by sleep breathing disorders [184,185].

Apelin favors the proliferation and progression of various cancers [186]. In a group of patients with diverse cancers, including lung cancer, gastrointestinal, breast, ovarian, uterus, and prostate cancers, apelin was proposed as a strong biomarker of cancer progression [187].

### 5.11. Retinol Binding Protein 4

Retinol binding protein 4 (RBP-4) is positively associated with obesity [188]. In OSA patients, increased plasma levels of RBP-4 were observed [189]. In another study, RBP-4 serum levels in OSA patients were not influenced by the severity of the disease and did not correlate with sleep disturbances but were significantly decreased during treatment with continuous positive airway pressure [190]. Serum RBP-4 levels were associated with sleep quality in pregnant women [191]. Sleep duration does not influence RBP-4 [139].

In some cancer types (e.g., breast cancer), increased RBP-4 plasma levels were found when compared to healthy controls and non-metastatic breast cancer patients [192].

### 5.12. Galectin-3

The expression of galectin-3 is upregulated in obese patients [193]. Serum galectin-3 levels in OSA were increased and correlated with the severity of this syndrome [194]. In another study, plasma galectin-3 levels were increased only in women with OSA [195]. In a study of patients with moderate to severe OSA, galectin-3 serum levels were not found to be a useful biomarker of the severity of the disease [196].

Galectin-3 contributes to the proliferation and progression of some cancers [197,198,199].

The summarization of the associations of adipokines and their pro- or anti-inflammatory properties with obesity, sleep disorders and cancer is presented in Table 1.

## 6. Final Remarks and Conclusions

Some of the peptides excreted by adipocytes and/or myocytes have pro-inflammatory proprieties (leptin, resistin, chemerin, vaspin, RBP-4 [202]) or anti-inflammatory proprieties (adiponectin, omentin, vaspin and irisin [202,214]). Cancer development and evolution may depend on chronic inflammation [215]; thus, an imbalance in the pro-inflammatory and anti-inflammatory effects of adipokines/adipomyokines may also be one of the factors influencing cancer development.

The presented review indicates that one of the possible pathways influencing the development of cancer may be the mutual relationship between obesity and/or sarcopenia, sleep quantity and quality, and adipokines/adipomyokines excretion. This indicates a tempting field for further studies on the associations of adipose tissue and skeletal muscles’ paracrine/endocrine function with sleep disturbances in cancer patients. Considering the high proportion of persons with obesity and sedentary lifestyles, as well as the associations of these conditions with sleep disturbances, more attention should be paid to the individual and combined effects on cancer pathophysiology.

## Figures and Tables

**Table 1:** The associations between adipokines/adipomyokines and their pro-inflammatory properties with obesity, sleep disorders and cancer.

Adipokines/ Adipomyokines	Level in Obesity	Level in Sleep Disorders	Pro-Inflammatory Properties	Role in Cancerogenesis
adiponectin	↓	↓	Yes [200] and no [201]	Anti-cancerous [119], decreased in many cancers [120,121,122].
omentin	↓	↓	No [202]	Anti-cancerous or pro-cancerous [122,126], increased in some cancers [127,129], decreased in some cancers [130,131,132]
leptin	↑	↑	Yes [202]	Pro-cancerous [140], increased in some cancers [141,142,143,144,145], controversial in some cancers [146,203]
resistin	↑	↑ or ↓ or =	Yes [202]	Increased in some cancers [149,150,151,152] or not changed in some cancers [153]
vaspin	↑	↑	No [202]	Increased in some cancers [156], decreased in some cancers [157]
chemerin	↑	↑	Yes [202] and no [204]	Anti-cancerous or pro-cancerous [161], increased in various cancers [203]
nesfatin	↑	↓ or =	No [205]	Increased in some cancers [168,169], decreased or not changed in some cancers [170]
visfatin	↑	↑	Yes [206]	Increased in various cancers [175]
osteopontin	↑	↑	Yes [207]	Increased in various cancers [179,203]
apelin	↑	↑ or =	No [208]	Pro-cancerous [186], increased in various cancers [187]
RBP-4	↑	↑ or =	Yes [209]	Increased in various cancers [192]
galectin-3	↑	↑ or ↓ or =	Yes [210]	Increased in various cancers [211,212]
irisin	↑ or ↓	↑ or ↓	No [213]	Anti-cancerous [89,96,97,98,99] or without pro-/anti-cancerous action [100], increased in some cancers [108,109], decreased in some cancers [102,105,106,107]

## Data Availability

This review is based on the PubMed publications.
